# Spatially varying effects of predictors for the survival prediction of nonmetastatic colorectal Cancer

**DOI:** 10.1186/s12885-018-4985-2

**Published:** 2018-11-08

**Authors:** Yu Tian, Jun Li, Tianshu Zhou, Danyang Tong, Shengqiang Chi, Xiangxing Kong, Kefeng Ding, Jingsong Li

**Affiliations:** 1 Engineering Research Center of EMR and Intelligent Expert System, Ministry of Education, Collaborative Innovation Center for Diagnosis and Treatment of Infectious Diseases, College of Biomedical Engineering and Instrument Science, Zhejiang University, No. 38 Zheda Road, Hangzhou, 310027 Zhejiang Province China; 2 Department of Surgical Oncology, Second Affiliated Hospital, Zhejiang University School of Medicine, No. 88 Jiefang Road, Hangzhou, 31009 Zhejiang Province China

**Keywords:** Colorectal cancer, SEER, TNM staging system, Survival prediction model, Spatially varying effects

## Abstract

**Background:**

An increasing number of studies have identified spatial differences in colorectal cancer survival. However, little is known about the spatially varying effects of predictors in survival prediction modeling studies of colorectal cancer that have focused on estimating the absolute survival risk for patients from a wide range of populations. This study aimed to demonstrate the spatially varying effects of predictors of survival for nonmetastatic colorectal cancer patients.

**Methods:**

Patients diagnosed with nonmetastatic colorectal cancer from 2004 to 2013 who were followed up through the end of 2013 were extracted from the Surveillance Epidemiology End Results registry (Patients: 128061). The log-rank test and the restricted mean survival time were used to evaluate survival outcome differences among spatial clusters corresponding to a widely used clinical predictor: stage determined by AJCC 7th edition staging system. The heterogeneity test, which is used in meta-analyses, revealed the spatially varying effects of single predictors. Then, considering the above predictors in a standard survival prediction model based on spatially clustered data, the spatially varying coefficients of these models revealed that some covariate effects may not be constant across the geographic regions of the study. Then, two types of survival prediction models (a statistical model and a machine learning model) were built; these models considered the predictors and enabled survival prediction for patients from a wide range of geographic regions.

**Results:**

Based on univariate and multivariate analysis, some prognostic factors, such as “TNM stage”, “tumor size” and “age at diagnosis,” have significant spatially varying effects among different regions. When considering these spatially varying effects, machine learning models have fewer assumption constraints (such as proportional hazard assumptions) and better predictive performance compared with statistical models. Upon comparing the concordance indexes of these two models, the machine learning model was found to be more accurate (0.898[0.895,0.902]) than the statistical model (0.732 [0.726, 0.738]).

**Conclusions:**

Based on this study, it’s recommended that the spatially varying effect of predictors should be considered when building survival prediction models involving large-scale and multicenter research data. Machine learning models that are not limited by the requirement of a statistical hypothesis are promising alternative models.

**Electronic supplementary material:**

The online version of this article (10.1186/s12885-018-4985-2) contains supplementary material, which is available to authorized users.

## Background

Colorectal cancer (CRC) is the most common gastrointestinal malignant tumor worldwide [1]. Although the overall mortality rate is low, the mortality rate is higher in developing countries, and significant differences in mortality have been observed among countries and regions [2].

An increasing number of studies, especially large-scale, multicenter population studies, have identified spatial differences in the survival of colorectal and other cancer patients [3–8]. Douaiher et al. noted significant differences in survival outcomes between developed and developing countries [9, 10]. Mokdad et al. [11] found significant differences in the mortality rate of CRC patients between different regions and counties in the United States, with the highest mortality rate in Union County, Florida (58.4/100000 people; 95% Confidence Interval: 52.0–65.2), and the lowest mortality rate in Summit County, Colorado (8.1/100000 people; 95% CI: 7.0–9.3). The decrease in mortality from 1980 to 2014 also varies among counties, with the largest decreases found in Howard County, Maryland (62.2%; 95% UI: 60.7–67.4%) and Nassau County, New York (62.3%; 95% UI: 60.1%, 64.3%) [11]. Michael et al. also reported regional differences in the management and outcomes of CRC patients in Australia [12].

In studies of survival prediction modeling for CRC based on large-scale and multicenter data aggregated from a wide range of geographic regions, little attention has been paid to the spatially varying effects of predictors [13–18]. Many studies have assumed that aggregated data are homogeneous and directly used statistical models, such as the Cox proportional hazard model, which assumes that all patients are independent, regardless of origin [19–22]. Some studies suggested that patients from the same geographic region were likely to have correlated outcomes, thus violating the assumption of independent observations. Therefore, researchers should consider a multilevel survival model for survival prediction, including a single random effect that considers similarities within spatial clusters [23–26]. All the regression coefficients of these models are assumed to have a constant impact across the entire study region, meaning that the impact of the patients’ characteristics remain constant among different geographic regions. However, recent research has shown that there are spatially varying effects of predictors in breast cancer survival prediction, indicating that the impact of patient characteristics on breast cancer survival varies by location [27].

This study aims to detect and interpret the spatially varying effect of predictors using population-based CRC survival data aggregated from a wide range of geographic regions. The studied predictors included the following widely used clinical predictors: tumor, node, metastasis (TNM) tumor stage; demographic factors; tumor differentiation grade; histological type; tumor location; tumor size; and number of positive regional lymph nodes. Overall survival was considered the outcome of interest. A machine learning model (random survival forest, RSF) was then developed. The model requires no statistical restrictions or assumptions to build a survival prediction model and can be used as an alternative survival prediction model to statistical models for multilevel survival when dealing with spatially varying effects of predictors.

## Methods

### Patients

This study obtained CRC patient data from the Surveillance, Epidemiology and End Results (SEER) program of the National Cancer Institute (NCI), which includes 18 population-based registries [28]. We regarded these registries as spatial clusters of patients and explored the spatially varying effect of predictors among these registries. Patient information, including demographics, diagnoses and survival, are routinely collected, and this information is publicly available as deidentified data.

**Fig. 1:**
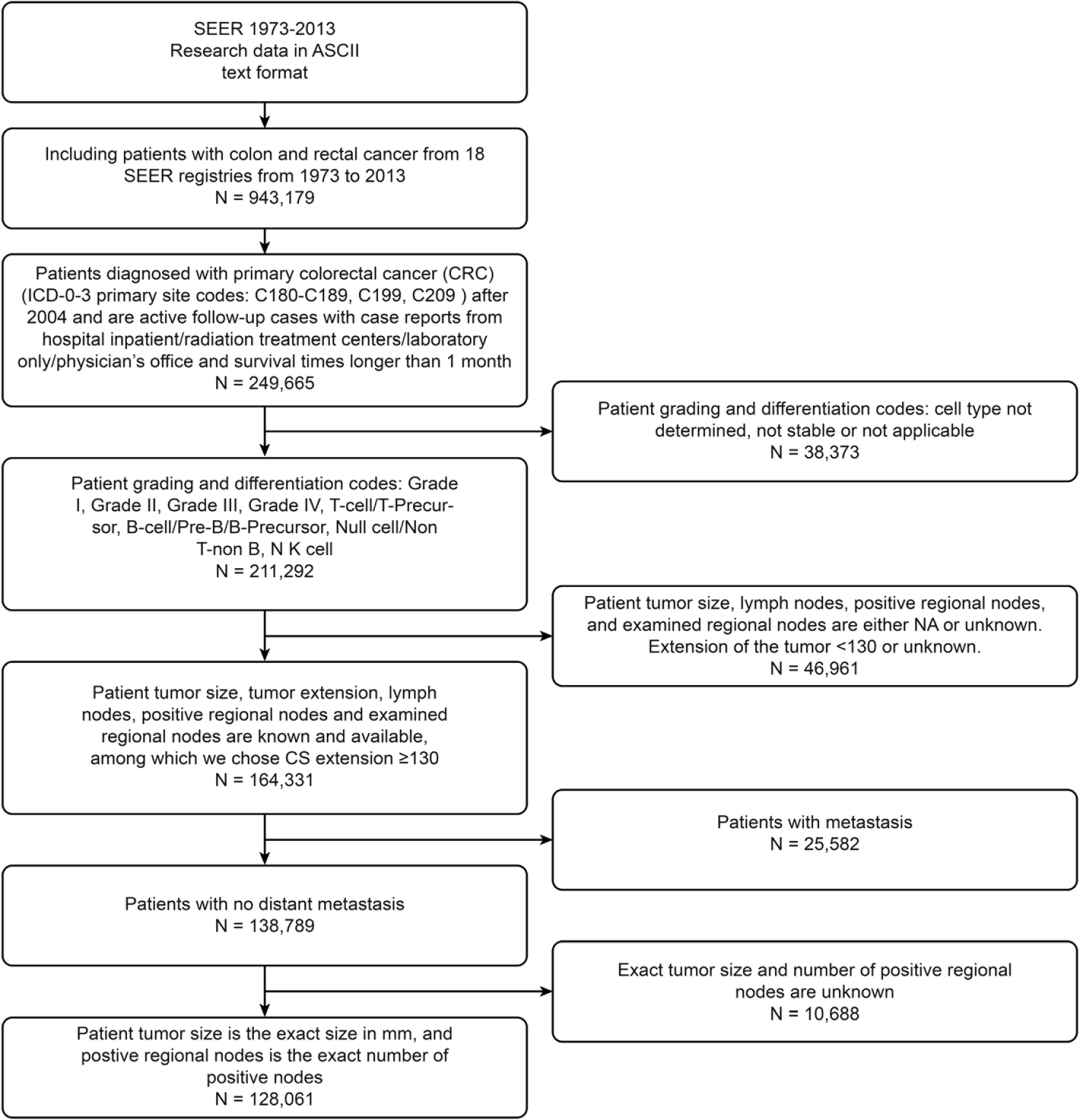
Details of the patient data screening procedure. For all SEER research data reported from 1973 to 2013, we first considered patients who were diagnosed with primary colorectal cancer after 2004 and who were actively followed-up with case reports from hospital inpatient departments, radiation treatment centers, laboratories, and physicians’ offices and for whom survival times were longer than 1 month; these criteria yielded 249,665 patients. We then excluded patients whose tumor grading and differentiation codes were of undetermined cell types or were not stable or not treated, reducing the number of patients to 211,292. We next excluded patients whose tumor size, lymph nodes, positive regional nodes and examined regional nodes were either NA or unknown, further reducing the sample to 164,331 patients. Finally, we excluded patients with metastases and patients for whom the exact tumor size and number of positive regional nodes were unknown. We ultimately obtained 128,061 patients from 18 SEER registries as our study cohort

In this study, the analysis was limited to patients who were diagnosed with primary nonmetastatic CRC (SEER primary site recodes C180-C189, C199 or C209 without distant metastasis) as their only malignant tumor and were actively followed up from January 2004 through December 2013. We excluded individuals if their cancer status was obtained from a nursing home, hospice, autopsy report or death certificate; if their survival time was less than 1 month; if their tumor size was not reported as an exact value; or if the number of cancer-positive regional lymph nodes was not noted. In addition, patients with unknown key predictor variables were excluded (Fig. 1).

### Univariate analysis

First, the spatial varying effect was detected for a widely used clinical predictor: the TNM staging system. In the TNM staging system, T represents the depth of primary tumor penetration, N represents the number of regional nodes involved in the tumor, and M represents distant metastasis. Based on these parameters, patients were divided into 7 groups that correspond to different prognoses: stage I, stage IIA, stage IIB, stage IIC, stage IIIA, stage IIIB and stage IIIC. The TNM staging system can be used as an independent criterion for distinguishing patients based on survival outcomes. Based on these criteria of the tumor stage, each patient was assigned a TNM staging label. Then, log-rank tests (the survdiff function in the “survival” R package [29]) were used to evaluate differences in the Kaplan-Meier survival curves among spatial clusters. A quantitative comparison based on the restricted mean survival time (RMST) (the rmst2 function in the “survRM2” R package [30]) was performed, which intuitively reflects the spatially varying effects of the TNM staging system.

Second, the heterogeneity test from the meta-analysis was adopted to reveal the spatially varying effects of the patient characteristics. First, univariate associations between overall survival and predictors in each spatial cluster were examined using a Cox regression model, from which log hazard ratios were obtained with 95% confidence intervals. Then, Cochran’s heterogeneity [31] statistic Q value and the inconsistency index I^2^ [32] were used to examine the heterogeneity of the predictors across the entire study region, revealing the spatially varying effects of the predictors.

### Multivariate analysis

The predictors listed in Table 1 were fitted using Cox regression models [33] based on the patient data from different spatial clusters, and regression coefficients were thereby obtained for predictors from different spatial clusters. For comparison and interpretation, we used “Age at diagnosis” as a category variable and defined the following groups: Group 1: less than 55 years old; Group 2: between 55 and 64 years old; Group 3: between 65 and 74 years old; and Group 4: older than 75 years. We compared the variance of the regression coefficient for each predictor among the spatial clusters, revealing the impact of predictors across the entire study region.

**Table 1:** Demographic and Clinical Characteristics of SEER Nonmetastatic CRC Patients from 2004 to 2013

Characteristic	Levels/IQR ^a^	Number (%)	Overall death (%)
Age at diagnosis	< 55	26,838 (20.96)	3763 (10.88)
	55–64	28,485 (22.24)	4917 (14.21)
	65–74	31,580 (24.66)	7474 (21.60)
	≥75	41,158 (32.14)	18,445 (53.31)
	IQR	67 [56,77]	
Gender	Male	63,617 (49.68)	17,089 (49.39)
	Female	64,444 (50.32)	17,510 (50.61)
Grade	Well differentiated	11,309 (8.83)	2435 (7.04)
	Moderately differentiated	92,992 (72.62)	23,472 (67.84)
	Poorly differentiated	21,189 (16.55)	7802 (22.55)
	Undifferentiated	2571 (2.01)	890 (2.57)
Histology	Adenocarcinoma	94,972 (74.16)	26,063 (75.33)
	Mucinous adenocarcinoma	11,523 (9.00)	3829 (11.07)
	Papillary adenocarcinoma	11,697 (9.13)	2366 (6.84)
	Adenoma. In Adenoma. Polyp	6525 (5.10)	1097 (3.17)
	Signet ring cell carcinoma	1071 (0.84)	516 (1.49)
	Other	2273 (1.77)	728 (2.10)
Tumor location^b^	Right colon	60,432 (47.19)	17,735 (51.26)
	Left colon	37,763 (29.49)	9471 (27.37)
	Rectum	29,866 (23.32)	7393 (21.37)
T stage (AJCC7)	T1	13,178 (10.29)	1843 (5.33)
	T2	23,017 (17.97)	4538 (13.11)
	T3	76,464 (59.71)	21,734 (62.82)
	T4a	8783 (6.86)	3411 (9.86)
	T4b	6619 (5.17)	3073 (8.88)
N stage (AJCC7)	N0	76,716 (59.91)	16,867 (48.75)
	N1a	15,089 (11.78)	4285 (12.38)
	N1b	15,027 (11.73)	4842 (13.99)
	N1c	787 (0.61)	110 (0.32)
	N1nos	2857 (2.23)	777 (2.25)
	N2a	9686 (7.56)	3778 (10.92)
	N2b	7872 (6.15)	3937 (11.38)
	N2nos	27 (0.02)	3 (0.01)
Tumor size	IQR	40 mm [30,60]	
EOD10_PN ^c^	IQR	0 [0,2]	
Registry	San Francisco-Oakland SMSA	6825 (5.33)	1738 (5.02)
	Connecticut	6116 (4.78)	1709 (4.94)
	Metropolitan Detroit	6542 (5.11)	1965 (5.68)
	Hawaii	2773 (2.17)	690 (2.00)
	Iowa	4053 (3.16)	839 (2.42)
	New Mexico	2759 (2.15)	725 (2.10)
	Seattle (Puget Sound)	4093 (3.20)	662 (1.91)
	Utah	2604 (2.03)	649 (1.88)
	Metropolitan Atlanta	4135 (3.23)	1013 (2.93)
	Alaska	252 (0.20)	61 (0.18)
	San Jose-Monterey	3255 (2.54)	745 (2.15)
	Los Angeles	13,604 (10.62)	3718 (10.75)
	Rural Georgia	299 (0.23)	91 (0.26)
	Greater California	28,557 (22.30)	7703 (22.26)
	Kentucky	8704 (6.80)	2488 (7.19)
	Louisiana	8131 (6.35)	2296 (6.64)
	New Jersey	15,152 (11.83)	4696 (13.57)
	Greater Georgia	10,207 (7.97)	2811 (8.12)

^a^*IQR*: Interquartile range and medians [1st Qu, 3rd Qu] were used to describe continuous variables

^b^Tumor location, the right colon comprised the cecum, appendix, ascending colon, and hepatic flexure; the left colon comprised the splenic flexure, descending colon, sigmoid colon, large intestine, and NOS; and the rectum comprised the rectosigmoid junction and the rectum

^c^*EOD10_PN*: Number of positive regional lymph nodes

### Survival prediction based on the statistical model and the machine learning model

In this paper, we apply a machine learning model (RSF [34]) to make full use of large-scale, multicenter clinical research data without violating the statistical assumptions requiring all patients to be independent of one another and requiring the impact of predictors to remain constant across the entire study region. The performances of the machine learning model and the statistical model (Cox proportional hazards model with mixed effects Cox, Accelerated Failure Time Model AFT) were then evaluated and compared based on testing data aggregated from multiple geographic regions. We used the concordance index (C-index) [35] and prediction error curves [36] as measures of the model’s prediction performance. The C-index is one of the most commonly used performance measures of survival models. It can be interpreted as the fraction of all pairs of subjects whose predicted survival times are correctly ordered among all subjects who can actually be ordered. To reflect the performance variance of both models among different spatial clusters, we compared the variance of the C-index for each model among the different spatial clusters. Prediction error curves can be used to show model calibration performance via an expected time-dependent *Brier score*. For correctly censored data, the squared residual (observed status-predicted status)^2^ of a subject at each particular time point t is weighted using the inverse probability of the censoring weights [37], which can yield the calibration ability of the prediction model within a certain follow-up period.

The modeling construction process is outlined in Additional file 1: Figure S1. First, we divided the selected SEER data based on region codes and then divided the data of each regional dataset into a training set (80%) and a test set (20%). Second, we fit all the training data to models based on the machine learning approach and the statistical approach and tested the prediction performance of both models using the test datasets for the different regions. The deduction process, including setting the model parameters and inputting the factors, is described in the Additional file 1.

All analyses were performed using R version 3.4.0.

## Results

### Patient demographics and characteristics

**Fig. 2:**
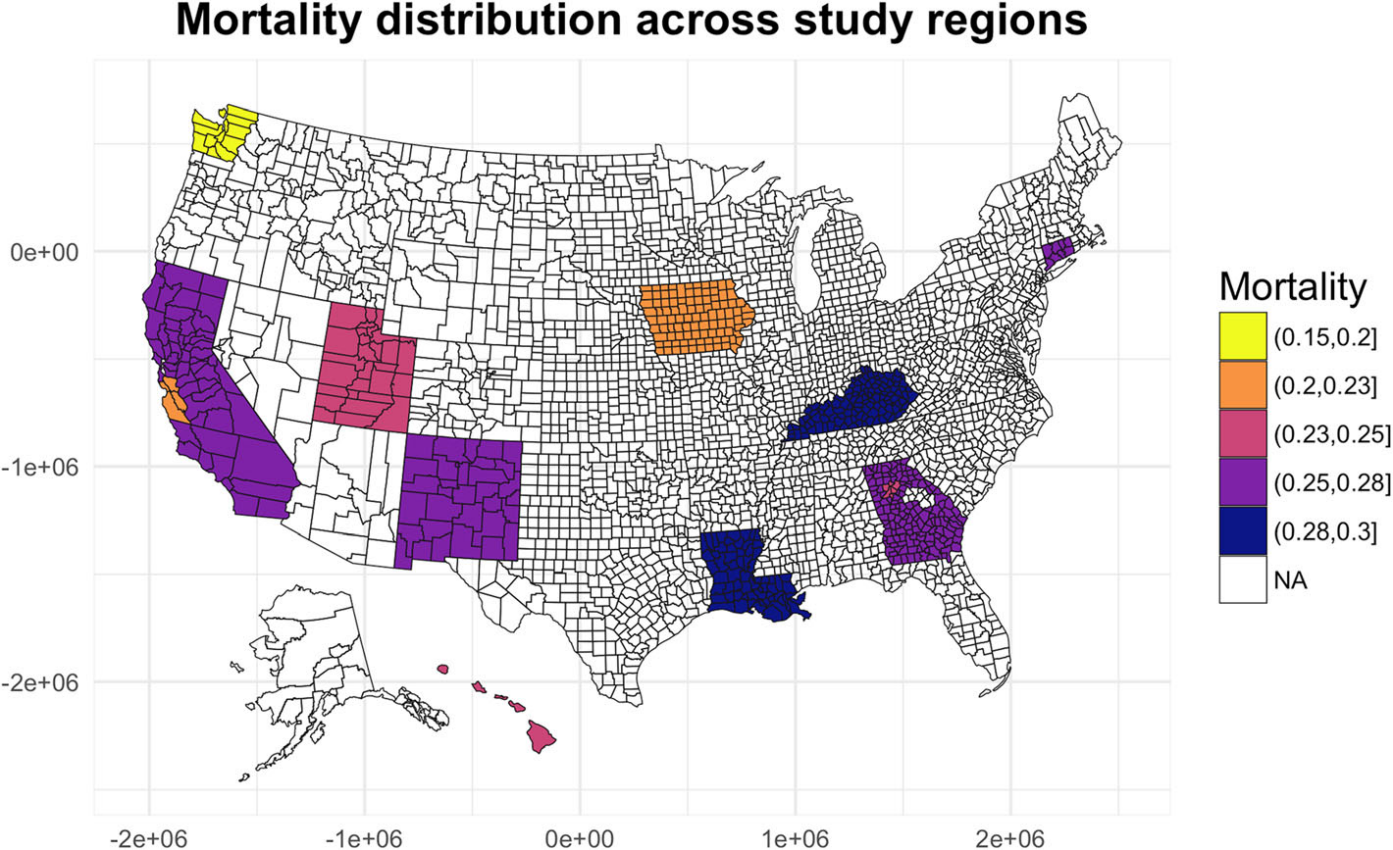
Mortality distribution across all study regions. Similar to previous findings, differences in survival outcomes across geographic areas were found in this study. The urban mortality rate was lower than the suburban mortality rate, and the mortality rate in areas with high levels of economic activity was lower than that in areas with low levels of economic activity

A total of 128,061 patients met the inclusion criteria and were included in the analysis. The overall percentage of excluded patients was 86.42%. The patient demographics and characteristics are listed in Table 1. The median follow-up time was 40 months (range, 1–119 months). Overall deaths were recorded for 34,599 (27.02%) patients. The median age at diagnosis was 67 years. Patients were categorized into age groups of less than 55 years, 55 to 64 years, 65 to 74 years and greater than 74 years. We considered age at diagnosis as a categorical factor in the multivariate analysis for comparison and interpretation purposes. However, we used age as a continuous variable in both the statistical and machine learning prediction models. Therefore, this parameter had both levels and interquartile range values. The median tumor size was 40 mm (interquartile range, 30 to 60; maximum, 975). The median number of positive regional lymph nodes was 0 (interquartile range, 0 to 2; maximum, 73). The probability of all-cause death varied across different geographic regions (Fig. 2). As mentioned in a previous study, the survival outcome of patients with CRC exhibits spatial cluster effects, which may impact the choice of methodology in building a survival prediction model.

### Spatially varying effects of the predictors according to univariate analysis

**Fig. 3:**
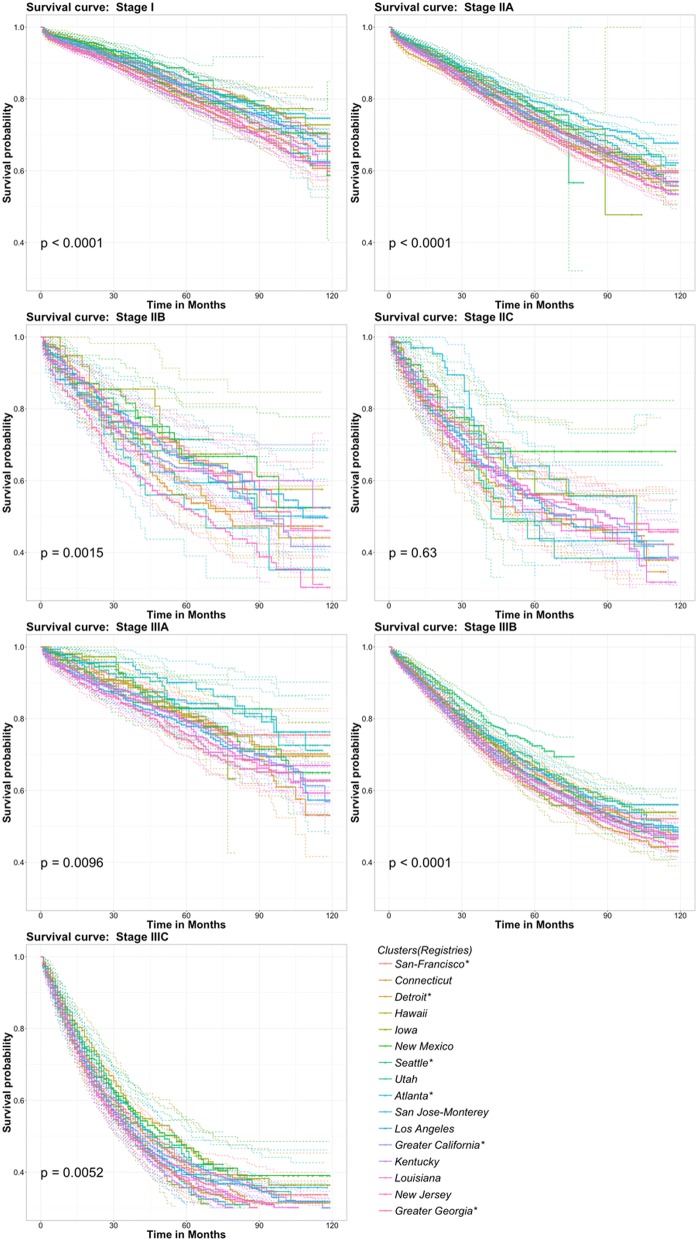
Kaplan-Meier survival curves of patients within the same TNM staging group across different spatial clusters. The log-rank test was used to test differences across different spatial clusters. Except for the stage IIC group, there were significant spatially varying effects (*P* value < 0.05) of patients within the same staging groups

For the widely used clinical predictor, the TNM tumor stage, we first compared the differences in survival outcomes for patients within the same TNM staging group among different spatial clusters. Because the “Alaska” and “Rural Georgia” clusters included too few patients [252 patients in Alaska (0.2% of the total population) and 299 patients in Rural Georgia (0.23% of the total population)], we drew Kaplan-Meier survival curves for all spatial clusters except these two clusters. As shown by the Kaplan-Meier survival curves (Fig. 3), the survival outcomes of patients within the same TNM staging group significantly differed across different spatial clusters. Except for the stage IIC group, there were significant spatially varying effects (*P* value < 0.05) for patients assigned to the same staging groups. Second, using the San Francisco-Oakland SMSA registry as a reference, we performed a quantitative comparison of patient survival outcomes within the same staging group among spatial clusters based on the RMST. As shown in Table 2, in most staging groups, the RMSTs of the spatial clusters significantly differed. For stage IIB, the maximum difference in RMST was approximately 15.66% of the entire assessment period, which means that for the same staging group, patients in different spatial clusters showed different survival outcomes.

**Table 2:** RMST of Different Clusters for Patients within the Same AJCC7 Stage Group

AJCC7 Stage group	RMST Max	RMST Min	RMSTSD^a^	Spatial Cluster [Max, Min] ^b^	Max-Diff	Max-Diff/Tau^c^
Stage I	88.49	82.22	2.00	[Seattle (Puget Sound), Kentucky]	6.28	6.28%
Stage IIA	68.96	64.48	1.29	[San Jose-Monterey, Metropolitan Detroit]	4.48	5.60%
Stage IIB	58.81	47.69	3.02	[Hawaii, New Jersey]	11.12	15.66%
Stage IIC	55.32	46.51	2.47	[New Mexico, Connecticut]	8.80	12.40%
Stage IIIA	71.92	63.56	2.15	[San Jose-Monterey, Greater Georgia]	8.36	10.86%
Stage IIIB	62.51	55.97	1.72	[Seattle (Puget Sound), Metropolitan Detroit]	6.54	8.61%
Stage IIIC	47.69	40.69	2.19	[Hawaii, Kentucky]	7.00	9.34%

^a^SD, standard deviation

^b^Spatial Cluster[Max, Min] indicates the registry with the maximum or minimum RMST

^c^Max-Diff/Tau indicates the proportion of the maximum difference in RMST among clusters within the entire assessment period (Tau)

**Fig. 4:**
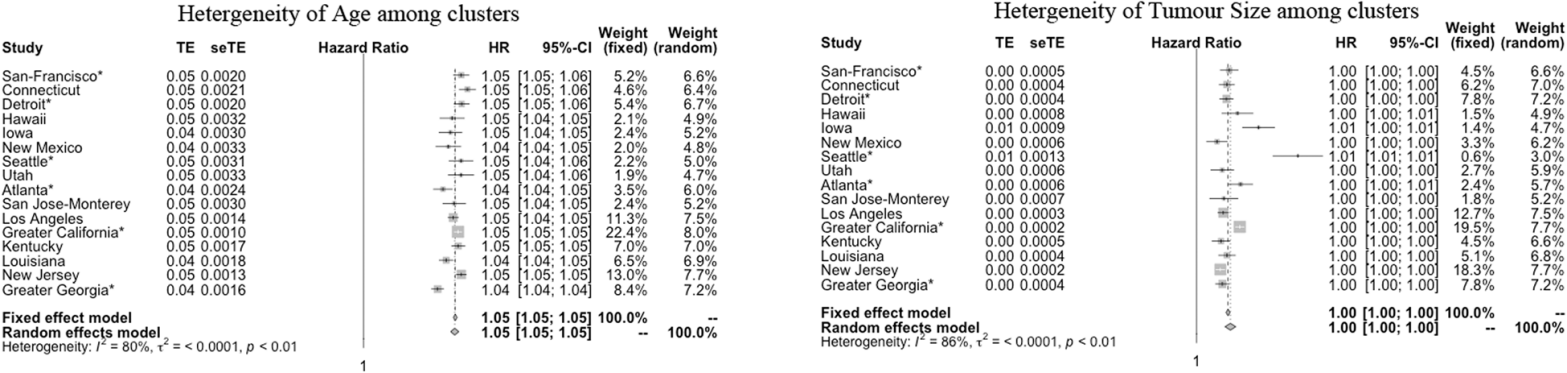
Forest plot displaying the spatially varying effects of “tumor size” and “age at diagnosis”

For other predictors that are usually considered in studies of survival prediction, we first analyzed the univariate associations between overall survival and the predictors in each spatial cluster using a Cox regression model from which log hazard ratios were obtained with 95% confidence intervals. Then, using Cochran’s heterogeneity statistic Q and the inconsistency index I^2^ as measurements, we obtained the heterogeneity of the impact of predictors on survival outcomes among different spatial clusters. As shown in Table 3, the effects of “tumor size,” “number of positive regional lymph nodes” and “age at diagnosis” on survival outcomes were substantially heterogeneous among the different spatial clusters, which indicated that the impact of the patients’ characteristics may not be consistent among different geographic regions. A forest plot was used to intuitively display the spatially varying effects for “tumor size” and “age at diagnosis” (Fig. 4).

**Table 3:** Heterogeneity Test for Predictors among Spatial Clusters

Covariate	Description	Q^a^	I^2 b^
Age	Age at diagnosis	73.74	79.66%
Gender	Male set to 0 as baseline hazard		
	Female	21.35	29.75%
Grade	Well-differentiated set to 0 as baseline hazard		
	Moderately differentiated	28.29	46.97%
	Poorly differentiated	42.21	64.47%
	Undifferentiated	25.96	42.23%
Histology	Adenocarcinoma set to 0 as baseline hazard		
	Mucinous adenocarcinoma	14.61	0.00%
	Papillary adenocarcinoma	17.13	12.42%
	Adenoma. In Adenoma. Polyp	22.32	32.81%
	Signet ring cell carcinoma	17.88	16.12%
	Other	14.28	0.00%
Tumor location	Right colon set to 0 as baseline hazard		
	Left colon	29.26	48.74%
	Rectum	20.01	25.05%
Tumor size	The size of the tumor	105.16	85.74%
EOD10_PN^c^	Number of positive lymph nodes	84.56	82.26%

^a^*Q*: Cochran’s heterogeneity statistic

^b^I2: Inconsistency index

^c^*EOD10_PN*: Number of positive regional lymph nodes

### The spatially varying effect of the regression coefficient on the multivariate analysis

**Fig. 5:**
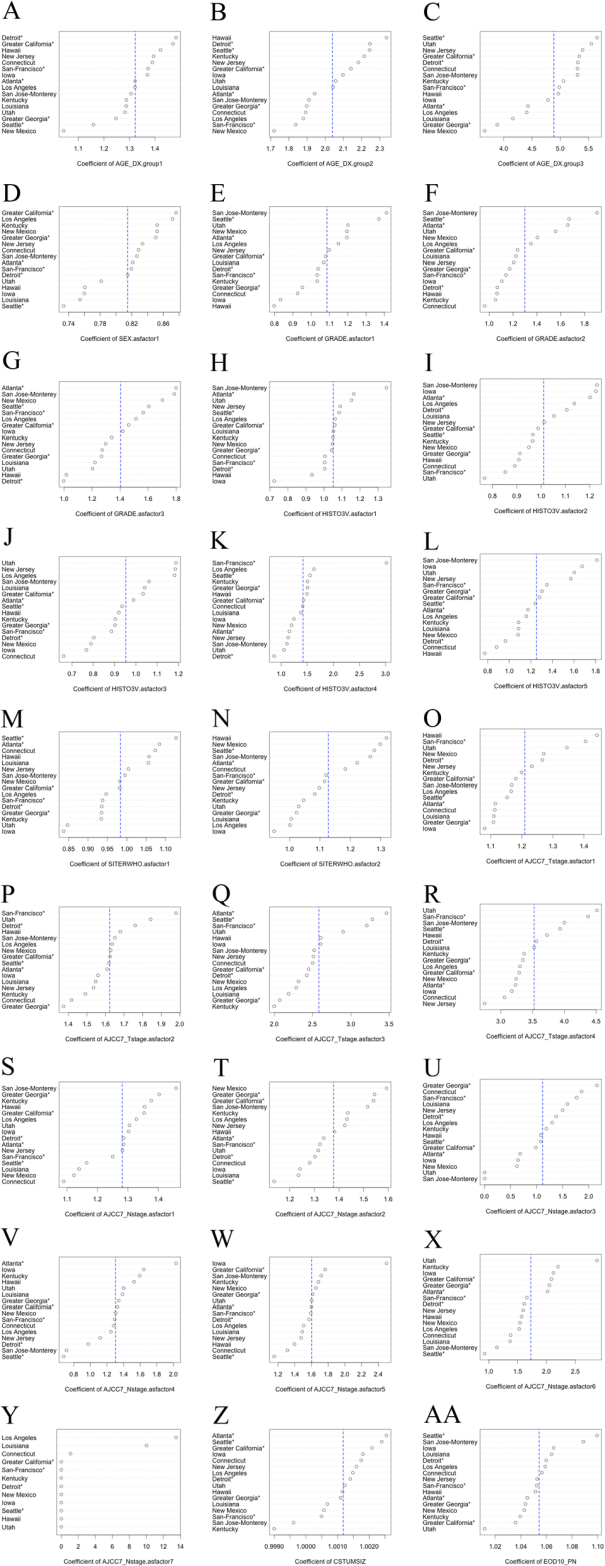
Spatially varying effects of the regression coefficients of predictors on the multivariable analysis. The predictors included **a**) age at diagnosis between 55 and 64 years; **b**) age at diagnosis between 65 and 74 years; **c**) age at diagnosis greater than or equal to 75 years; **d**) female; **e**) moderately differentiated tumor grade; **f**) poorly differentiated tumor grade; **g**) undifferentiated tumor grade; **h**) mucinous adenocarcinoma histology type; **i**) papillary adenocarcinoma histology type; **j**) adenoma. In adenoma, polyp histology type; **k**) signet ring cell carcinoma histology type; **l**) other histology type; **m**) left colon; **n**) rectum; **o**) T stage T2; **p**) T stage T3; **q**) T stage T4a; **r**) T stage T4b; **s**) N stage N1a; **t**) N stage N1b; **u**) N stage N1c; **v**) N stage N1nos; **w**) N stage N2a; **x**) N stage N2b; **y**) N stage N2nos; **z**) tumor size; and AA) number of positive regional lymph nodes

**Fig. 6:**
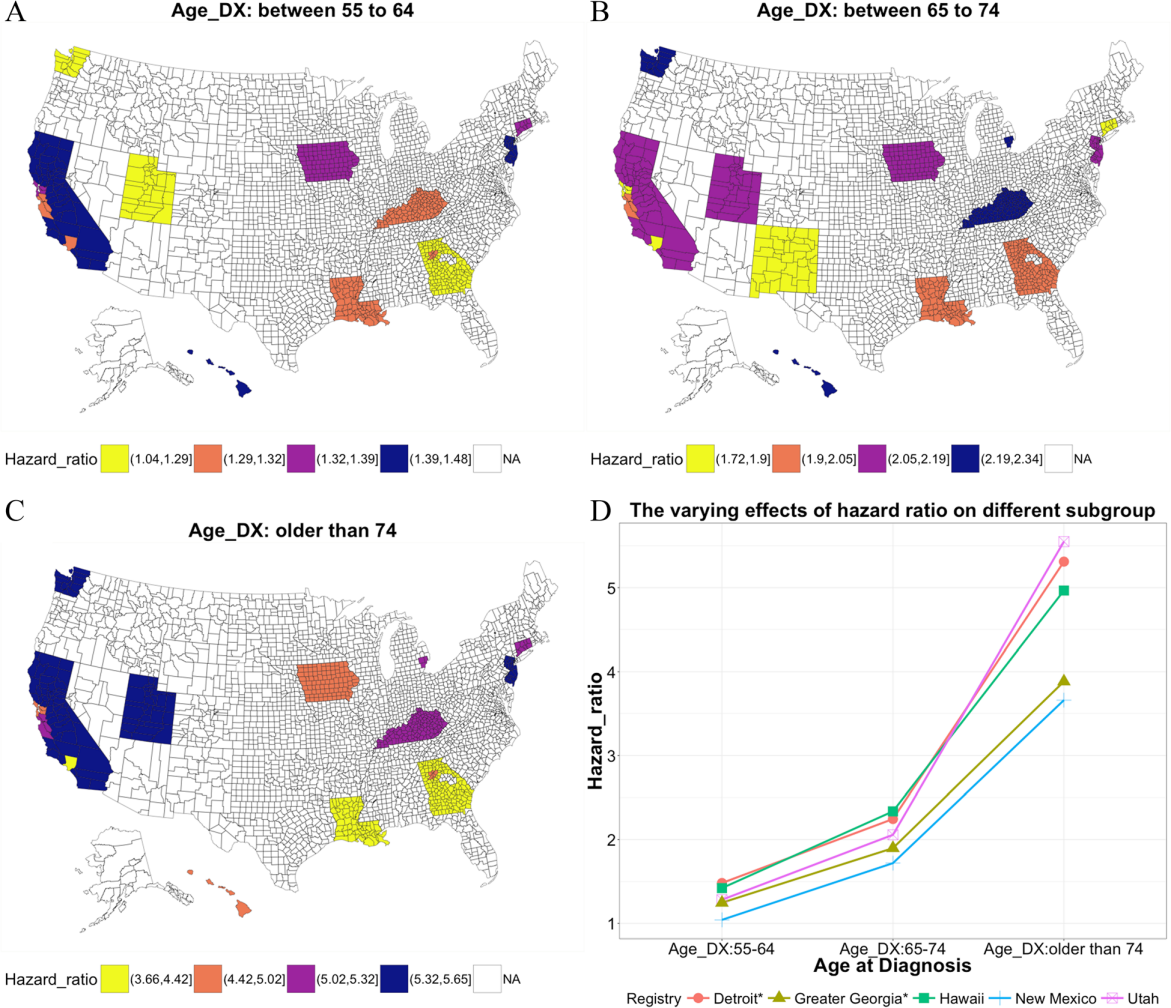
Hazard ratios according to “age at diagnosis” group and registry. **a**) Subgroup 1: age at diagnosis between 55 and 64 years, **b**) Subgroup 2: age at diagnosis between 65 and 74 years, **c**) Subgroup 3: age at diagnosis greater than or equal to 75 years. **d**) The varying effects of hazard ratio on different subgroups of registries, including Detroit, Greater Georgia, Hawaii, New Mexico and Utah

Considering the regression coefficients of the predictors combined through multivariate Cox regression models among the different spatial cluster datasets, we found strong spatially varying effects of the regression coefficient. We considered male patients, age at diagnosis less than 55 years, well-differentiated tumor, adenocarcinoma histology type, right colon tumor location, T1 T stage and N0 N stage as the baseline hazard. As shown in Fig. 5, the pattern of the spatial variation of the regression coefficient differed among predictors; the spatially varying effect of “age at diagnosis” was large, but spatially varying effects for “tumor size” and “number of positive regional lymph nodes” were relatively small. The pattern of spatially varying effects also differed among the subgroups of one predictor. For the predictor “histological type,” the regression coefficients of the subgroups of “mucinous adenocarcinoma” and “signet ring cell carcinoma” among the different spatial clusters were more stable than those of the other subgroups of “histological type.” As shown in more detail in Fig. 6, the impact of the same predictors (e.g., “age at diagnosis”) on different geographic regions also varied, and the hazard ratio (the exponential transformation of the regression coefficient of the predictor) of “Hawaii” remained higher than that of “Greater Georgia” and “New Mexico” within all the subgroups. However, for other registries, these patterns may not exist. For example, the hazard ratio of “Detroit” for the subgroup of patients with age at diagnosis = 55–64 years was similar to that of “Hawaii” for the same subgroup but lower than that of “Hawaii” for the age at diagnosis = 65–74 years subgroup and much higher than that of “Hawaii” for the age at diagnosis > 74 years subgroup.

In summary, we found that the predictors commonly used in survival prediction models have significant spatially varying effects and that the impact of patient characteristics may not remain constant across large-scale, multicenter clinical research data that have been aggregated from a wide range of geographic regions.

### Model evaluation and comparison

**Fig. 7:**
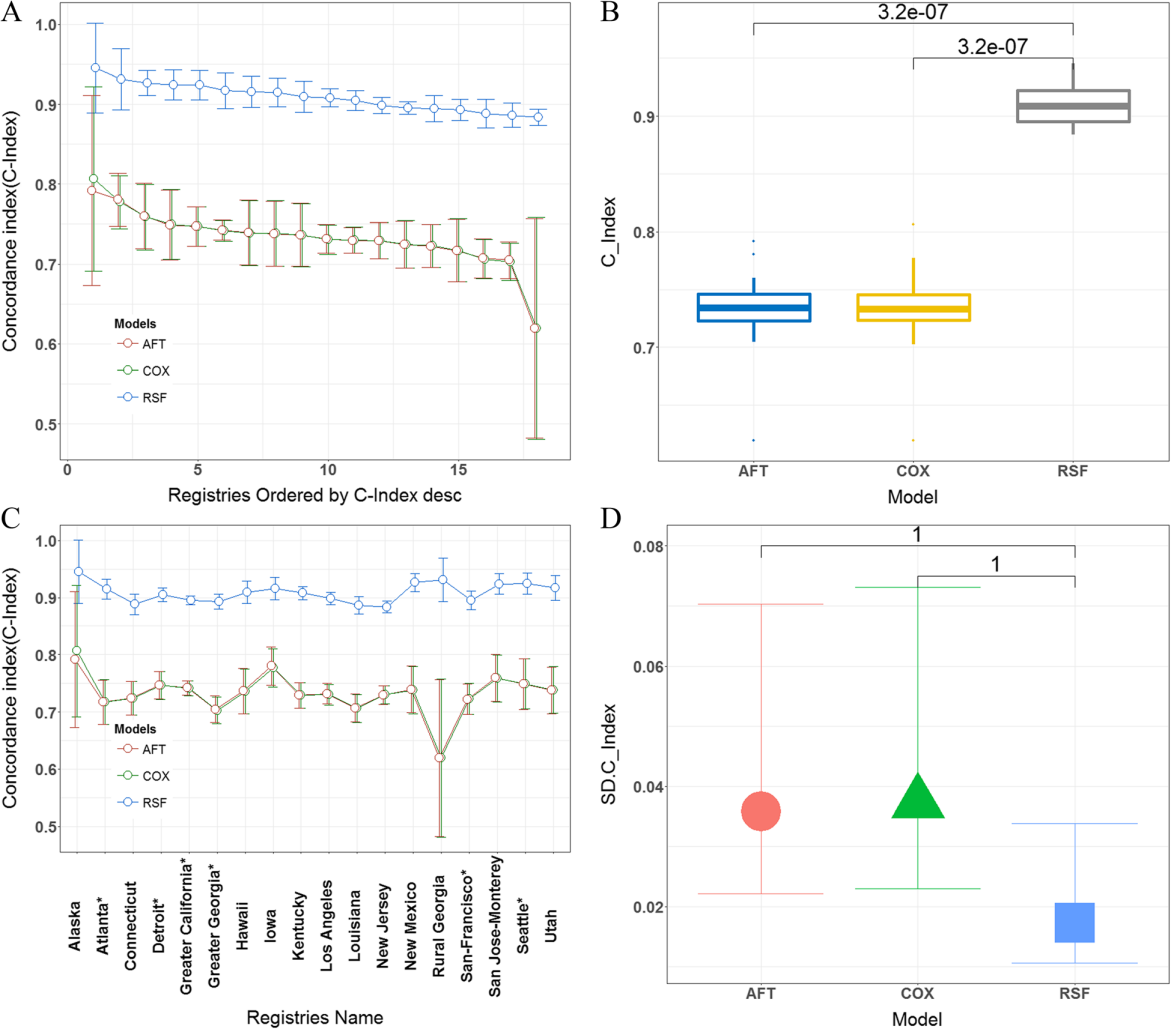
Model evaluation and comparison between the machine learning model and the statistical model. **a**) C-indexes of both models among different spatial clusters sorted by decreasing C-indexes. **b**) Boxplot of C-indexes of both models among different spatial clusters using the Wilcoxon test (nonparametric) to compare the mean C-indexes of the models. **c**) The C-indexes of both models among different spatial clusters. **d**) Comparison of the standard deviations of the C-indexes of both models among different spatial clusters using the Wilcoxon test (nonparametric). The machine learning model exhibited better prediction performance than the statistical model

**Fig. 8:**
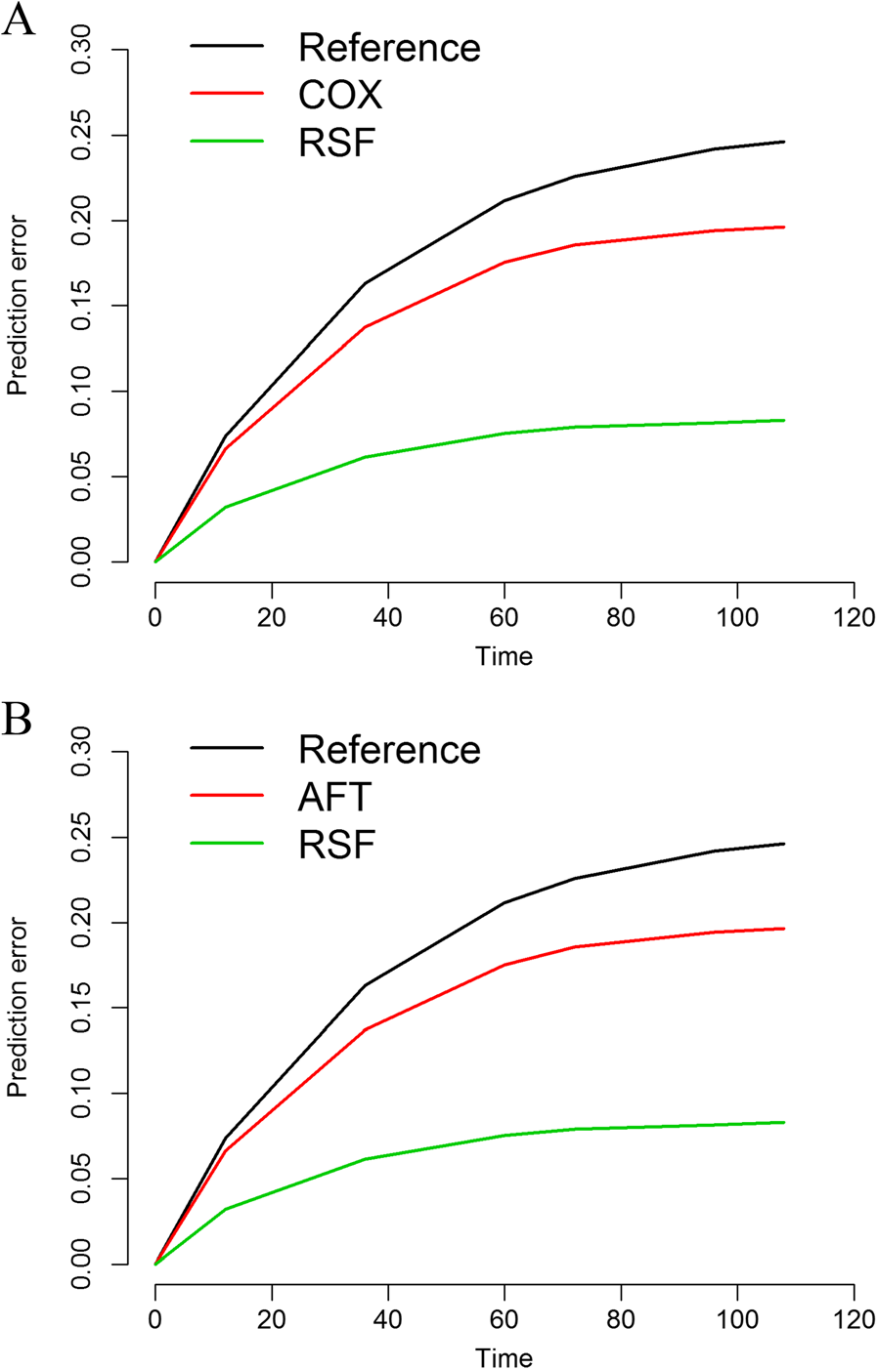
Model calibration performance based on prediction error curves. The nonparametric Kaplan-Meier curve was used as a reference. Cox and AFT represent the statistical model, and RSF represents the machine learning model. The prediction error was estimated at 0, 12, 36, 60, 72, 96, and 108 months after diagnosis. As the prediction error curves of Cox and AFT were nearly overlapping, we plotted their curves separately. **a**) Prediction error curves for Cox and RSF. **b**) Prediction error curves for AFT and RSF

The predictive accuracies of the machine learning model and the statistical model were measured using the C-index. The stability of both models for different spatial cluster datasets was evaluated based on the standard deviation and the difference between the maximum and minimum of the C-index for different spatial cluster datasets. As shown in Table 4, the machine learning model exhibited better prediction performance for the test dataset than the statistical model. As shown in Additional file 1: Table S5, the proportional hazard assumption for the Cox model was violated and may affect the reliability of the model; therefore, we consider the AFT model as an alternative strategy for the analysis of time-to-event data. The AFT model can even be suitable when hazards are not proportional. The C-index for the machine learning model (0.898, 95% CI: [0.895, 0.902]) was higher than that for the AFT model (0.732, 95% CI: [0.726, 0.738]). Although there were no significant differences (*P* = 1) in the model’s prediction stability for different spatial cluster datasets, the RSF model had a lower standard deviation and lower maximum and minimum differences of the C-index. As shown in Fig. 7, the prediction accuracy of both models for different spatial cluster datasets also varied, especially for regions that contained fewer patients. The RSF model yielded a higher prediction accuracy with a narrower error bar for the C-index (95% confidence interval) than the AFT model, indicating that the RSF model was more accurate and stable for different spatial cluster datasets that contained a different number of patients and predictors with spatially varying effects. As shown in Fig. 8, the prediction error of both models was tested using an aggregate test dataset, and the reference model was a nonparametric Kaplan-Meier curve. The RSF model consistently had a lower prediction error than the statistical model and, therefore, had better model calibration capability than the statistical model. In addition, the statistical model had lower prediction error than the nonparametric method.

**Table 4:** Performance Comparison of the Statistical Model and the Machine Learning Model

Performance measurement	Model		Random survival forest
	Cox regression model with mixed effects	AFT model	
C-index on global dataset			
Test dataset	0.731 [0.725, 0.737]	0.732 [0.726, 0.738]	0.898 [0.895, 0.902]
C-index among different spatial clusters			
Standard deviation	0.037	0.035	0.017
Max-Min difference	0.187	0.172	0.062
Reduced SD^a^	reference	−5.41%	−54.05%
Reduced Max-Min^b^	reference	−8.02%	−66.84%

^a^*Reduced SD*: Reduction in the standard deviation of the C-index for the machine learning model compared with the statistical model

^b^*Reduced Max-Min*: Reduction in the Max-Min difference of the machine learning model compared with the statistical model

## Discussion

In this study, we used population-based data from the SEER database to detect and interpret the spatially varying effects of patients’ clinicopathological and demographic characteristics, which are commonly used in CRC survival prediction. The study period was between 2004 and 2013, a period during which patients benefited from modern therapies with improved survival probability. A new population-based survival prediction model is needed to predict CRC patient survival probabilities, as the impact of patient characteristics may not remain constant across entire study regions, especially for large-scale, multicenter clinical research, for which data are collected from a wide range of geographic regions. Strong spatially varying effects were identified for commonly used CRC predictors. To our knowledge, this study is the first to explore the spatially varying effects of the predictors used in a CRC survival prediction model with a population-based dataset. The machine learning model, which considered the varying impact of patient characteristics on different spatial clusters, achieved more accurate prediction than the statistical model, which considered only the random effects of spatial clustering and that the impact of patient characteristics remained constant across different spatial clusters of patients diagnosed with primary nonmetastatic CRC.

The spatially varying effects of predictors for CRC survival prediction were detected, while many previous studies have ignored these effects. TNM tumor staging, which is widely used worldwide for predicting cancer prognosis, assumes that patients in different geographic regions should have the same or similar survival outcomes based on the same pathological criteria. However, in our study, all stage groups except stage IIC exhibited significant variance in survival outcomes (*P* < 0.05). Therefore, using the TNM staging system to predict survival may potentially reveal deviations between different regions. Moreover, age at diagnosis also has spatially varying effects that are typically not considered. Using heterogeneity testing and multivariable regression analysis, we observed that age at diagnosis had large spatially varying effects. Compared with patients in Hawaii, patients from Utah had lower hazard ratios in subgroups for which the patients’ age at diagnosis ranged from 55 to 64 and from 65 to 74, but these hazard ratios were higher in the subgroup for which the patients’ age at diagnosis was greater than 74. However, for patients from Greater Georgia and New Mexico, the hazard ratios were consistently lower than those of patients in Hawaii for all subgroups related to age at diagnosis.

The spatially varying effects of the predictors imply that the impact of patients’ characteristics may not remain constant across entire study regions. The reasons for these effects should be studied further. However, many studies that have constructed survival prediction models using large-scale, multicenter clinical research data aggregated from a wide range of geographic regions did not consider these effects. Because these models assume that the impact of patient characteristics remains constant across different spatial clusters and consider only random effects regarding the spatial nature of the data, the use of these models may have over- or underestimated survival prediction. We achieved better performance using a machine learning model (RSF) that considered the spatially varying effect of predictors than when we used a statistical model.

In combination with our previous research results [38], the present study demonstrates that the RSF model can be used to study complex relationships (such as nonlinear or time-dependent relationships) regarding the problem of prognosis in nonmetastatic CRC; this topic warrants continued in-depth study. Using the proposed machine learning model framework, one can establish a global survival prediction model based on large-scale, multicenter clinical data that considers the spatially varying effects of predictors among different clusters. Once the model is finalized, patients from different regions will be able to obtain more personalized survival predictions by entering their individual characteristics. Both doctors and patients will benefit from such a model. Doctors will be able to provide more precise and generalized survival predictions and chose more appropriate treatment options, and patients will be able to better understand the progression of their disease, thus enhancing patient compliance.

However, we did not include therapeutic and molecular data, which might have further improved the predictive accuracy of the model. Moreover, we assumed that all patient treatments and clinical visits were confined to the same region because migration and cross-regional clinical visits were outside the scope of this study. Another limitation of this study is that the longest follow-up in the SEER database was only 119 months, and the median follow-up was 40 months; these follow-up times are relatively short considering a population with a potentially curable condition. Therefore, the results should be verified using other databases containing long-term follow-up data.

## Conclusions

We conclude that the widely used clinical TNM tumor staging system is limited by spatially varying effects for predicting survival. The impact of age at diagnosis, tumor grade, histology and tumor location may not be consistent across study regions. Constructing survival prediction models based on population-based data collected from a wide range of geographic regions without considering these spatially varying effects may produce deviations across different regions. Machine learning models that consider these spatially varying effects are likely to produce more accurate and robust survival prediction models.

## Additional file

Additional file 1: Supplementary material. (DOCX 266 kb)

## Abbreviations

AFT: accelerated failure time model
AJCC: American Joint Committee on Cancer
CI: confidence interval
C-index: concordance index
CRC: colorectal cancer
RMST: restricted mean survival time
RSF: random survival forest
SEER: Surveillance, Epidemiology, and End Results
TNM: tumor-node-metastasis

## References

[1] Miller KD, Siegel RL, Lin CC, Mariotto AB, Kramer JL, Rowland JH (2016). Cancer treatment and survivorship statistics, 2016. CA Cancer J Clin.

[2] Ferlay J, Soerjomataram I, Dikshit R, Eser S, Mathers C, Rebelo M (2015). Cancer incidence and mortality worldwide: sources, methods and major patterns in GLOBOCAN 2012. Int J Cancer.

[3] Tervonen HE, Morrell S, Aranda S, Roder D, You H, Niyonsenga T (2017). The impact of geographic unit of analysis on socioeconomic inequalities in cancer survival and distant summary stage–a population-based study. Aust N Z J Public Health.

[4] Swede H, Sarwar A, Magge A, Braithwaite D, Cook LS, Gregorio DI (2016). Mortality risk from comorbidities independent of triple-negative breast cancer status: NCI-SEER-based cohort analysis. Cancer Causes Control.

[5] Sleightholm R, Foster JM, Smith L, Ceelen W, Deraco M, Yildirim Y (2016). The American Society of Peritoneal Surface Malignancies multi-institution evaluation of 1,051 advanced ovarian cancer patients undergoing cytoreductive surgery and HIPEC: an introduction of the peritoneal surface disease severity score. J Surg Oncol.

[6] Liu Z, Zhang K, Du XL (2016). Risks of developing breast and colorectal cancer in association with incomes and geographic locations in Texas: a retrospective cohort study. BMC Cancer.

[7] Liang PS, Mayer JD, Wakefield J, Ko CW (2017). Temporal trends in geographic and sociodemographic disparities in colorectal cancer among medicare patients, 1973-2010. J Rural Health.

[8] Feng X, Tan X, Alenzi EO, Rai P, Chang W (2016). Spatial and temporal variations of screening for breast and colorectal cancer in the United States, 2008 to 2012. Medicine.

[9] Douaiher J, Ravipati A, Grams B, Chowdhury S, Alatise O, Are C (2017). Colorectal cancer-global burden, trends, and geographical variations. J Surg Oncol.

[10] Senthil M, Trisal V, Paz IB, Lai LL (2010). Prediction of the adequacy of lymph node retrieval in colon cancer by hospital type. Arch Surg.

[11] Mokdad AH, Dwyer-Lindgren L, Fitzmaurice C, Stubbs RW, Bertozzi-Villa A, Morozoff C (2017). Trends and patterns of disparities in cancer mortality among US counties, 1980-2014. JAMA.

[12] Ireland MJ, March S, Crawford-Williams F, Cassimatis M, Aitken JF, Hyde MK (2017). A systematic review of geographical differences in management and outcomes for colorectal cancer in Australia. BMC Cancer.

[13] Dalton ARH (2017). Incomplete diagnostic follow-up after a positive colorectal cancer screening test: a systematic review. J Public Health.

[14] Short PF, Moran JR, Yang TC, Camacho F, Gusani NJ, Mackley HB (2016). Effects of hospital type and distance on lymph node assessment for colon cancer among metropolitan and nonmetropolitan patients in Appalachia. Med Care Res Rev.

[15] Panchal JM, Lairson DR, Chan W, Du XL (2016). Geographic variation in oxaliplatin chemotherapy and survival in patients with colon cancer. Am J Ther.

[16] Ho V, Ku-Goto MH, Zhao H, Hoffman KE, Smith BD, Giordano SH (2016). Regional differences in recommended cancer treatment for the elderly. BMC Health Serv Res.

[17] Fournel I, Bourredjem A, Sauleau EA, Cottet V, Dejardin O, Bouvier AM (2016). Small-area geographic and socioeconomic inequalities in colorectal tumour detection in France. Eur J Cancer Prev.

[18] Kong X, Li J, Cai Y, Tian Y, Chi S, Tong D (2018). A modified TNM staging system for non-metastatic colorectal cancer based on nomogram analysis of SEER database. BMC Cancer.

[19] Shin A, Joo J, Yang HR, Bak J, Park Y, Kim J (2014). Risk prediction model for colorectal cancer: National Health Insurance Corporation study, Korea. PLoS One.

[20] Gabriel E, Attwood K, Thirunavukarasu P, Al-Sukhni E, Boland P, Nurkin S (2016). Predicting individualized postoperative survival for stage II/III colon cancer using a mobile application derived from the national cancer data base. J Am Coll Surg.

[21] Hippisley-Cox J, Coupland C (2017). Development and validation of risk prediction equations to estimate survival in patients with colorectal cancer: cohort study. BMJ.

[22] Watanabe T, Miyata H, Konno H, Kawai K, Ishihara S, Sunami E (2017). Prediction model for complications after low anterior resection based on data from 33,411 Japanese patients included in the National Clinical Database. Surgery.

[23] Austin PC (2017). A tutorial on multilevel survival analysis: methods, models and applications. Int Stat Rev.

[24] Crowther MJ, Look MP, Riley RD (2014). Multilevel mixed effects parametric survival models using adaptive gauss-Hermite quadrature with application to recurrent events and individual participant data meta-analysis. Stat Med.

[25] Dasgupta P, Cramb SM, Aitken JF, Turrell G, Baade PD (2014). Comparing multilevel and Bayesian spatial random effects survival models to assess geographical inequalities in colorectal cancer survival: a case study. Int J Health Geogr.

[26] Charvat H, Remontet L, Bossard N, Roche L, Dejardin O, Rachet B (2016). A multilevel excess hazard model to estimate net survival on hierarchical data allowing for non-linear and non-proportional effects of covariates. Stat Med.

[27] Hsieh CF, Cramb SM, Mcgree JM, Dunn NAM, Baade PD, Mengersen KL (2016). Does geographic location impact the survival differential between screen- and interval-detected breast cancers?. Stoch Env Res Risk A.

[28] SEER. http://www.seer.cancer.gov.

[29] Therneau T. A package for survival analysis in S. 2015. https://cran.r-project.org/web/packages/survival/index.html. Accessed 6 June 2017 2017.

[30] Uno H, Tian L, Cronin A, Battioui C, Horiguchi M. survRM2: comparing restricted mean survival time. 2015. https://cran.r-project.org/web/packages/survRM2/index.html. Accessed 10 June 2017.

[31] Higgins JP, Thompson SG (2002). Quantifying heterogeneity in a meta-analysis. Stat Med.

[32] Higgins JP, Thompson SG, Deeks JJ, Altman DG (2003). Measuring inconsistency in meta-analyses. BMJ.

[33] Andersen PK, Gill RD (1982). Cox's regression model for counting processes: a large sample study. Ann Stat.

[34] Ishwaran H, Kogalur UB. RandomForestSRC: random forests for survival, regression and classification (RF-SRC). 2016. https://cran.r-project.org/web/packages/randomForestSRC/index.html. Accessed 10 June 2017.

[35] Harrell FE, Lee KL, Califf RM, Pryor DB, Rosati RA (1984). Regression modelling strategies for improved prognostic prediction. Stat Med.

[36] Steyerberg EW, Vickers AJ, Cook NR, Gerds T, Gonen M, Obuchowski N (2010). Assessing the performance of prediction models: a framework for some traditional and novel measures Epidemiol (Camb MA). Epidemiology.

[37] Weathers B, Cutler R. Comparision of Survival Curves Between Cox Proportional Hazards, Random Forests, and Conditional Inference Forests in Survival Analysis. All Graduate Plan B and other Reports. 2017; 927. https://digitalcommons.usu.edu/gradreports/927.

[38] Chi S-Q, Tian Y, Li J, D-y T, Kong X-X, Poston G (2017). Time-dependent and nonlinear effects of prognostic factors in nonmetastatic colorectal cancer. Cancer Med.

