# Supplementary material for: Spatially varying effects of predictors for the survival prediction of nonmetastatic colorectal Cancer
Source: BMC Cancer. 2018 Nov 8;18:1084. doi: 10.1186/s12885-018-4985-2 (PMC6225720; doi:10.1186/s12885-018-4985-2)
Supplement: Supplementary file 1 — Supplementary material. (DOCX 266 kb) [file 12885_2018_4985_MOESM1_ESM.docx]

**Supplementary Material**

**Methods**

1. To explore the cluster effect of survival outcomes based on the AJCC 7^th^ edition staging system, we evaluated differences in the Kaplan-Meier survival curves among 16 clusters (2 clusters with very small numbers of patients were removed) based on log-rank tests [1]. The log-rank test is the most well-known and widely used test for such analyses. In this study, we questioned whether the registry groups differ among patients within each staging group. Therefore, we considered the log-rank test for P groups.

Suppose we observe data from P different groups, and the data from group p (p=1 …, P) are:

$$\left( X_{p1},\delta_{p1} \right)\ldots(X_{pn_{p}},\delta_{pn_{p}})$$

We construct a ($P\times2$) table at each of the K distinct times of death and compare the death rates among the P groups, conditional on the number at risk.

Let $t_{1}\ldots t_{K}$ represent the K ordered, distinct death times. $d_{pj}$ is the number of deaths in groups p at the j-th death time, and $r_{pj}$ is the number at risk at that time.

Let $\mathbf{O}_{j}={(d_{1j}\cdots d_{(P-1)j})}^{T}$ be a vector of the observed number of failures in groups 1 to (P-1), respectively, at the j-th death time. Given the risk sets $r_{1j}\cdots r_{Pj}$ and the fact that there are $d_{j}$ deaths, $\mathbf{O}_{j}$ has a distribution similar to a multivariate version of the hypergeometric function. $\mathbf{O}_{j}$ has a mean:

$$\mathbf{E}_{j}={(\frac{d_{j}r_{1j}}{r_{j}}\cdots\frac{d_{j}r_{(P-1)j}}{r_{j}})}^{T}$$

and a variance covariance matrix:

$\mathbf{V}_{j}=\left( \begin{matrix} v_{11j} \\ \begin{matrix} v_{12j} \\ \begin{matrix} \cdots\\ \cdots\end{matrix} \end{matrix} \end{matrix}\begin{matrix} v_{12j} \\ \begin{matrix} v_{22j} \\ \begin{matrix} \cdots\\ \cdots\end{matrix} \end{matrix} \end{matrix}\begin{matrix} \cdots\cdots\\ \begin{matrix} \cdots\cdots\\ \cdots\cdots\end{matrix} \end{matrix}\begin{matrix} v_{1(P-1)j} \\ \begin{matrix} v_{2(P-1)j} \\ \begin{matrix} \ldots\\ v_{(P-1)(P-1)j} \end{matrix} \end{matrix} \end{matrix} \right)$

where the l-th diagonal element is:

$$v_{llj}=r_{lj}(r_{j}-r_{lj})d_{j}(r_{j}-d_{j})/[r_{j}^{2}(r_{j}-1)]$$

The m-th off-diagonal element is:

$$v_{lmj}=r_{lj}r_{mj}d_{j}(r_{j}-d_{j})/[r_{j}^{2}(r_{j}-1)]$$

The resulting $\chi^{2}$ test for a single ($P\times1$) table would have (P-1) degrees and is constructed as follows:

$${(\mathbf{O}_{j}-\mathbf{E}_{j})}^{T}V_{k}^{-1}(\mathbf{O}_{j}-\mathbf{E}_{j})$$

$\mathbf{O}_{j}$, $\mathbf{E}_{j}$ and $\mathbf{V}_{j}$ can be replaced with the sums over the K distinct death times. That is, let$\mathbf{O}=\sum_{j=1}^{k} \mathbf{O}_{j}$, $\mathbf{E}=\sum_{j=1}^{k} \mathbf{E}_{j}$ and$\mathbf{V}=\sum_{j=1}^{k} \mathbf{V}_{j}$. Then, the test statistic is:

$${\mathbf{(O-E})}^{T}\mathbf{V}^{-1}(\mathbf{O-E})$$

In summary, in our study, the P-value of the log-rank test for one staging group is less than 0.05, which suggests that there are regional differences for this staging group.

2. We performed a quantitative comparison of patients within the same staging group among the 16 clusters based on the restricted mean survival time (RMST). Between-group summary metrics based on the RMST are useful alternatives to the hazard ratio or other model-based measures [2].

The RMST is defined as the area under the curve of the survival function up to time $\tau(<\infty)$

$\mu_{\tau}=\int_{0}^{\tau} S(t)dt$

where S(t) is the survival function of a time-to-event variable of interest. The interpretation of the RMST is that “When we follow up patients for $\tau$, patients will survive for $\mu_{\tau}$ on average” which is quite straightforward and a clinically meaningful summary of the censored survival data.

3. We performed a heterogeneity analysis of patients’ individual characteristics in different clusters [3]. Consider Cochran’s Q statistic, a chi-square ($\chi^{2}$) test of heterogeneity with k-1 degrees of freedom. We denote an estimate of parameter $\theta_{i}$ from study i (i=1, …, k) as $y_{i}$(in this study, these parameters represent the coefficient of covariance or the log hazard ratio of different covariates) and its precision (defined as the reciprocal of the estimate’s variance) as $w_{i}$. A summary estimate $\mu$ is calculated as a weighted average of the study estimates using the precisions as:

$$\hat{\mu}=\sum w_{i}y_{i}/\sum w_{i}$$

A test of the homogeneity of $\theta_{i}$is performed by referring to the statistic:

$$Q=\sum w_{i}{(y_{i}-\hat{\mu})}^{2}$$

The heterogeneity of individual characteristics among registries is better quantified using the inconsistency index$I^{2}$, which describes the percentage of total variation across regions that is due to heterogeneity rather than chance.

$I^{2}=100\%\times(Q-df)/Q$

where Q is Cochran’s heterogeneity statistic and df is the degrees of freedom. Negative values of$I^{2}$ are set equal to zero, so $I^{2}$ lies between 0% and 100%. A value of 0% indicates no observed heterogeneity, and larger values show increasing heterogeneity. A value >75% may be considered substantial heterogeneous [4].

4. Cox model with mixed effects (the shared frailty model)

When observations are clustered into groups such as hospitals or cities, the shared gamma frailty model is the most often adapted model. To deal with the spatially varying effects of predictors for the survival prediction, the shared frailty model was used instead of the standard Cox proportion hazard model as a reference model in this study. For the j-th patient of the i-th register, let $T_{ij}$ denote the event time under study, let $C_{ij}$ be the right-censoring time, and let $L_{ij}$ be the left truncation time. The observations $Y_{ij}$ are equal to min($T_{ij},C_{ij}$), and the censoring indicators are $\delta_{ij}=I\{Y_{ij}=T_{ij}\}$. The event times may be left-truncated and right-censored. The hazard function for a shared frailty model is:

$\lambda_{ij}\left( t | v_{i} \right)=v_{i}\lambda_{0}\left( t \right)\exp\left( \beta^{T}X_{ij} \right)=v_{i}\lambda_{ij}\left( t \right)$
where $\lambda_{0}\left( t \right)$ is the baseline hazard function, $X_{ij}$ is the covariate vector associated with the vector of regression parameters $\beta$, and $v_{i}$ is the random effect associated with the i-th register. We assume that the $v_{i}$ are independently and identically distributed from a gamma distribution with E($v_{i}$)=1 and Var($v_{i}$)=$\theta$,

i.e., $v_{i}\sim\Gamma(\frac{1}{\theta},\frac{1}{\theta})$. We observe $Y_{ij}$, $L_{ij}$ and $\delta_{ij}$. The full marginal log-likelihood for this model has an analytical formulation:

$$l\left( \phi\right)=\sum_{i=1}^{G} \left\{ \left[ \sum_{j=1}^{n_{i}} \delta_{ij}ln\lambda_{ij}\left( Y_{ij} \right) \right]-\left( \frac{1}{\theta}+m_{i} \right)ln\left[ 1+\theta\sum_{j=1}^{n_{i}} \Lambda_{ij}\left( Y_{ij} \right) \right] \right.$$

$$+\frac{1}{\theta}\ln\left[ 1+\theta\sum_{j=1}^{n_{i}} \Lambda_{ij}\left( L_{ij} \right) \right]+I_{\{m_{i}\neq0\}}\sum_{k=1}^{m_{i}} ln(1+\theta(m_{i}-k))$$

The covariates considered in the Cox modeling process are shown in Table S1.

5. Accelerated Failure Time (AFT) Model

Like the Cox proportional hazard model, the proportional hazards (PH) model is routinely employed to analyze time-to-event data in medical research. However, if the assumption of PH is violated, the results from a PH model will be difficult to generalize to situations where the length of follow-up is different from that used in the analysis. It is also difficult to translate the results into the effect upon the expected median duration of disease for a patient in a clinical setting.

The AFT approach is an alternative strategy for analyzing time-to-event data and can be suitable even when hazards are not proportional. The AFT model describes the relationship between survival probabilities and a set of covariates. The log-linear form of the AFT model with respect to time is given by:

$$\log T_{i}=\mu+\alpha_{1}X_{1i}+\alpha_{2}X_{2i}+\ldots+\alpha_{p}X_{pi}+\sigma\varepsilon_{i}$$

where $\mu$ is the intercept, $\sigma$ is a scale parameter, and $\varepsilon_{i}$ is a random variable that is assumed to have a particular distribution. For each distribution of $\varepsilon_{i}$, there is a corresponding distribution for T. The distribution of T includes Exponential, Weibull, Log-logistic, Log-normal, and Gamma.

The AFT models are fitted using the maximum likelihood method.

We can use statistical tests or statistical criteria to compare all these AFT models. The Akaike information criterion (AIC) can be used, which is defined as:

$$AIC=-2l+2(k+c)$$

where l is the log-likelihood, k is the number of covariates in the model, and c is the number of model-specific ancillary parameters.

Lower values of the AIC suggest a better model.

6. Random survival forest (RSF) model

To integrate regional differences in the prognosis model for patients with CRC, we proposed a data-driven model based on an RSF and comparing its prediction performance with the Cox proportional hazard model [5]. In this section, we describe in detail how the RSF was applied to integrate the patient characteristics [6].

RSFs are extensions of random forests for right-censored survival data. An RSF is a collection of randomly grown survival trees. Each tree is grown using an independent bootstrap sample of the learning data using random feature selection at each node. RSF trees are generally grown very deeply with many terminal nodes (the ends of the tree). Trees in competing risk forests are similarly grown, and the estimated values calculated within the terminal nodes define the ensemble. The procedures used to build an RSF for competing risks are as follows:

1. Draw B bootstrap samples from the learning data.
2. Grow a competing risk tree for each bootstrap sample. At each node of the tree, randomly select M (M < p) candidate variables. The node is split using the candidate variable that maximizes a competing risk splitting rule.
3. Grow the tree to full size under the constraint that a terminal node should have no less than n > 0 unique cases.
4. Calculate the cumulative incidence function (CIF), the cumulative event-specific hazard function, the mortality for every event in each tree and the event-free survival in each tree.
5. Take the average of each estimator over the B trees to obtain its ensemble.

For our analysis, we used log-rank randomization as the splitting rule, 300 trees and 4 candidate variables for every RSF model.

The covariates considered in the RSF modeling process are shown in Table S2.

**Results:**

1. Univariate analysis

Univariate associations between overall survival and predictors in each spatial cluster were examined using a Cox regression model. For each of the predictors, including **Age at diagnosis, Gender, Grade, Histology, Tumor location, Tumor size,** and **EOD10_PN**, we trained a univariate Cox regression model on different spatial clusters using the following code:

uni.local =coxph(Surv(Time,Status)~uni.predictor)

Then, we performed a meta-analysis to observe the spatially varying effects for different predictors. The overall summary results are shown in Table 3.

1. Multivariate analysis

The predictors, including **Age at diagnosis, Gender, Grade, Histology, Tumor location, T stage, N stage, Tumor size,** and **EOD10_PN**, were fitted in a Cox regression model based on patient data from different spatial clusters for the multivariate survival analysis to specifically study the spatially varying effects of the above predictors on different spatial clusters.

For comparison and interpretation, we used “Age at diagnosis” as a category variable with the following groups: Group 1: less than 55 years old, Group 2: between 55 and 64 years old, Group 3: between 65 and 74 years old, and Group 4: older than 75 years old.

The Cox regression formulae:

multi.local=coxph(Surv(Time,Status)~Age_DX.asfactor+Gender.asfactor+Grade.asfactor

+ Histology.asfactor+Tumorlocation.asfactor+Tstage.asfactor+Nstage.asfactor

+TumorSize+EOD10_PN)

As shown in Table S3, the regression coefficients for each covariate differed among spatial clusters.

1. Statistical model based on a Cox model with mixed effects

After analyzing the spatially varying effects of predictors, this study compared two models that could account for the spatially varying effects of predictors. The input factors for both models are shown in Table S1 and Table S2.

The model formulae for the statistical model based on a Cox model with mixed effects were as follows:

mod.cox.train=coxph(Surv(Time,Status)~frailty(Spatial cluster ID)

+Age_DX +Gender.asfactor+Grade.asfactor

+ Histology.asfactor+Tumorlocation.asfactor+Tstage.asfactor+Nstage.asfactor

+TumorSize+EOD10_PN, data=traindata)

This model was trained on the patient data aggregated from all the spatial clusters. The regression coefficients of the covariates for the statistical model are shown in Table S4, and the results of the proportional hazards assumption test are shown in Table S5. Based on these results, the proportional risk of many covariates changes over time, which violates the hypothetical premise of the Cox model, namely the proportional hazard assumption. Therefore, we consider alternative statistical modeling approaches that do not have this constraint.

1. Statistical model based on the AFT model

Based on the results of the proportional hazards assumption test shown in Table S5, the proportional hazards assumptions were violated, so we consider the AFT model as an alternative strategy to analyze time-to-event data because it can be suitable even when hazards are not proportional.

The model formulae for the statistical model based on the AFT model were as follows:

mod.aft.train=psm(Surv(srv_time_mon,Status)~ frailty(Spatial cluster ID)

+AGE_DX+SEX.asfactor+GRADE.asfactor

+Histology.asfactor+Tumorlocation.asfactor+Tstage.asfactor+Nstage.asfactor

+TumorSize+EOD10_PN, data=traindata, dist = distribution)

We used AIC to choose the most suitable distribution for our dataset, and as shown in Table S6, we chose the log-logistic distribution for the final AFT model, which was used for patient survival prediction as an alternative approach when the PH assumption was violated.

References

1. Therneau TM, Grambsch PM. Modeling survival data: extending the Cox model. Technometrics*.* 2002;44:85-6.

2. Uno H, Tian L, Cronin A, Battioui C, Horiguchi M. survRM2: Comparing restricted mean survival time. 2015. <https://cran.r-project.org/web/packages/survRM2/index.html>. Accessed 10 June 2017.

3. Schwarzer G, Carpenter JR, Rücker G. Meta-analysis with R. Use R; 2015.

4. Haidich AB. Meta-analysis in medical research. Hippokratia*.* 2010;14:29-37.

5. Cox DR. Regression models and life-tables. J R Stat Soc*.* 1972;34:187-220.

6. Ishwaran H, Kogalur UB. randomSurvivalForest: Random Survival Forests; 2012.

**Table S1.** Covariates considered in the Cox modeling process.

| Covariate | Description | Levels |
| --- | --- | --- |
| Age | Age at diagnosis | Continuous variable |
| Gender | Gender | Male |
|  |  | Female |
| Grade | Tumor grade | Well differentiated |
|  |  | Moderately differentiated |
|  |  | Poorly differentiated OR Undifferentiated |
| Histology | Tumor histology | Adenocarcinoma group |
|  |  | Mucinous adenocarcinoma |
|  |  | Papillary adenocarcinoma |
|  |  | Adenoma. In Adenoma. Polyp |
|  |  | Signet ring cell carcinoma |
|  |  | Others |
| Site | Tumor location | Right colon |
|  |  | Left colon |
|  |  | Rectum |
| T stage | T refers to the size and extent of the primary tumor (AJCC 7) | T1 |
|  |  | T2 |
|  |  | T3 |
|  |  | T4a |
|  |  | T4b |
| N stage | N refers to the number of nearby lymph nodes that have cancer (AJCC 7) | N0 |
|  |  | N1a |
|  |  | N1b |
|  |  | N1c |
|  |  | N1nos |
|  |  | N2a |
|  |  | N2b |
|  |  | N2nos |
| Tumor size | Size of tumor | Continuous variable |
| EOD10_PN | Number of positive regional lymph nodes | Continuous variable |
| Spatial cluster | Population-based register | **Frailty term** |

**Table S2.** Covariates considered in the RSF modeling process.

| Covariate | Description | Levels |
| --- | --- | --- |
| Age | Age at diagnosis | Continuous variable |
| Gender | Gender | Male |
|  |  | Female |
| Grade | Tumor grade | Well differentiated |
|  |  | Moderately differentiated |
|  |  | Poorly differentiated OR Undifferentiated |
| Histology | Tumor histology | Adenocarcinoma group |
|  |  | Mucinous adenocarcinoma |
|  |  | Papillary adenocarcinoma |
|  |  | Adenoma. In Adenoma. Polyp |
|  |  | Signet ring cell carcinoma |
|  |  | Others |
| Site | Tumor location | Right colon |
|  |  | Left colon |
|  |  | Rectum |
| T stage | T refers to the size and extent of the primary tumor (AJCC 7) | T1 |
|  |  | T2 |
|  |  | T3 |
|  |  | T4a |
|  |  | T4b |
| N stage | N refers to the number of nearby lymph nodes that have cancer (AJCC 7) | N0 |
|  |  | N1a |
|  |  | N1b |
|  |  | N1c |
|  |  | N1nos |
|  |  | N2a |
|  |  | N2b |
|  |  | N2nos |
| Tumor size | Size of tumor | Continuous variable |
| EOD10_PN | Number of positive regional lymph nodes | Continuous variable |
| Spatial cluster | 18 population-based registers | **Individual factor** |

Table S3a. The regression coefficients of covariates among different spatial clusters.

| Predictor | Levels | San Francisco-Oakland SMSA | | Connecticut | Metropolitan Detroit | | Hawaii | Iowa | New Mexico | Seattle | Utah |
| --- | --- | --- | --- | --- | --- | --- | --- | --- | --- | --- | --- |
| Age at diagnosis | Age at diagnosis <55 set to 0 as baseline hazard | | | | | | | | | | |
|  | 55-64 | | 0.29 | 0.37 | | 0.42 | 0.39 | 0.22 | 0.23 | 0.25 | 0.41 |
|  | 65-74 | | 0.55 | 0.71 | | 0.82 | 0.91 | 0.63 | 0.67 | 0.94 | 0.81 |
|  | ≥75 | | 1.60 | 1.72 | | 1.70 | 1.70 | 1.50 | 1.37 | 1.77 | 1.76 |
| Gender | Male set to 0 as baseline hazard | | | | | | | | | |  |
|  | Female | | -0.18 | -0.19 | | -0.20 | -0.29 | -0.34 | -0.10 | -0.31 | -0.21 |
| Grade | Well-differentiated group set to 0 as baseline hazard | | | | | | | | | |  |
|  | Moderately differentiated | | 0.05 | -0.08 | | 0.01 | -0.11 | -0.08 | 0.11 | 0.35 | 0.13 |
|  | Poorly differentiated | | 0.09 | -0.03 | | 0.05 | 0.20 | 0.16 | 0.39 | 0.55 | 0.42 |
|  | Undifferentiated | | 0.41 | 0.27 | | -0.04 | -0.06 | 0.37 | 0.37 | 0.36 | 0.13 |
| Histology | Adenocarcinoma group set to 0 as baseline hazard | | | | | | | | | | |
|  | Mucinous adenocarcinoma | | -0.06 | -0.02 | | -0.03 | -0.13 | -0.28 | -0.04 | 0.03 | 0.12 |
|  | Papillary adenocarcinoma | | -0.12 | -0.15 | | 0.11 | 0.13 | 0.18 | -0.11 | 0.05 | -0.21 |
|  | Adenoma. In Adenoma. Polyp | | -0.16 | -0.35 | | -0.13 | -0.08 | -0.25 | -0.03 | 0.03 | 0.14 |
|  | Signet ring cell carcinoma | | 1.15 | 0.42 | | -0.02 | 0.43 | 0.27 | 0.05 | 0.81 | -0.38 |
|  | Other | | 0.37 | 0.23 | | -0.11 | -0.42 | 0.52 | 0.07 | 0.24 | 0.42 |
| Tumor location | Right colon set to 0 as baseline hazard | | | | | | | | | | |
|  | Left colon | | -0.08 | 0.09 | | -0.05 | 0.03 | -0.24 | -0.05 | 0.10 | -0.23 |
|  | Rectum | | 0.14 | 0.20 | | 0.12 | 0.33 | 0.00 | 0.26 | 0.22 | 0.02 |
| T stage | T1 set to 0 as baseline hazard | | | | | | | | | | |
|  | T2 | | 0.30 | 0.10 | | 0.17 | 0.35 | -0.01 | 0.25 | 0.24 | 0.40 |
|  | T3 | | 0.63 | 0.32 | | 0.48 | 0.55 | 0.35 | 0.57 | 0.54 | 0.68 |
|  | T4a | | 1.11 | 0.88 | | 0.82 | 0.99 | 0.88 | 0.96 | 1.19 | 0.98 |
|  | T4b | | 1.39 | 1.08 | | 1.12 | 1.42 | 0.98 | 1.23 | 1.32 | 1.59 |
| N stage | N0 set to 0 as baseline hazard | | | | | | | | | | |
|  | N1a | | 0.15 | 0.09 | | 0.17 | 0.37 | 0.34 | 0.11 | 0.18 | 0.34 |
|  | N1b | | 0.25 | 0.20 | | 0.28 | 0.30 | 0.22 | 0.42 | 0.19 | 0.25 |
|  | N1c | | -0.47 | 0.86 | | 0.12 | 0.39 | -0.21 | -0.41 | 0.14 | -13.18 |
|  | N1nos | | 0.24 | 0.13 | | 0.07 | 0.34 | 0.39 | 0.26 | -0.76 | 0.26 |
|  | N2a | | 0.51 | 0.29 | | 0.48 | 0.33 | 0.98 | 0.42 | 0.16 | 0.28 |
|  | N2b | | 0.61 | 0.23 | | 0.46 | 0.43 | 0.69 | 0.26 | 0.03 | 0.59 |
|  | N2nos | | NA | 0.06 | | -11.00 | -12.78 | -12.09 | -11.42 | -12.38 | -14.48 |
| Tumor size | Mean value as baseline hazard | | 0.00 | 0.00 | | 0.00 | 0.00 | 0.00 | 0.00 | 0.00 | 0.00 |
| EOD10_PN | Mean value as baseline hazard | | 0.05 | 0.06 | | 0.06 | 0.05 | 0.07 | 0.05 | 0.10 | 0.06 |

Table S3b. The regression coefficients of covariates among different spatial clusters.

| Predictor | Levels | Metropolitan Atlanta | San Jose-Monterey | Los Angeles | Greater California | Kentucky | | Louisiana | New Jersey | Greater Georgia |
| --- | --- | --- | --- | --- | --- | --- | --- | --- | --- | --- |
| Age at diagnosis | Age at diagnosis <55 set to 0 used as baseline hazard | | | | | | | | | |
|  | 55-64 | 0.19 | 0.23 | 0.30 | 0.41 | 0.30 | 0.28 | | 0.28 | 0.19 |
|  | 65-74 | 0.67 | 0.64 | 0.65 | 0.79 | 0.80 | 0.74 | | 0.76 | 0.64 |
|  | ≥75 | 1.48 | 1.66 | 1.52 | 1.68 | 1.64 | 1.43 | | 1.68 | 1.35 |
| Gender | Male set to 0 as baseline hazard | | | | | | | | | |
|  | Female | -0.17 | -0.23 | -0.13 | -0.13 | -0.17 | -0.30 | | -0.17 | -0.17 |
| Grade | Well-differentiated group set to 0 as baseline hazard | | | | | | | | | |
|  | Moderately differentiated | 0.18 | 0.27 | 0.15 | 0.09 | 0.02 | 0.11 | | 0.10 | -0.04 |
|  | Poorly differentiated | 0.48 | 0.57 | 0.34 | 0.22 | 0.10 | 0.28 | | 0.19 | 0.18 |
|  | Undifferentiated | 0.64 | 0.75 | 0.56 | 0.29 | 0.27 | 0.28 | | 0.28 | 0.18 |
| Histology | Adenocarcinoma group set to 0 as baseline hazard | | | | | | | | | |
|  | Mucinous adenocarcinoma | 0.09 | 0.31 | 0.07 | 0.08 | 0.07 | 0.06 | | 0.10 | 0.03 |
|  | Papillary adenocarcinoma | 0.16 | 0.22 | 0.13 | 0.01 | 0.02 | 0.05 | | 0.05 | -0.11 |
|  | Adenoma. In Adenoma. Polyp | -0.03 | 0.10 | 0.20 | 0.04 | -0.04 | -0.05 | | 0.21 | -0.17 |
|  | Signet ring cell carcinoma | 0.20 | 0.12 | 0.46 | 0.50 | 0.35 | 0.30 | | 0.19 | 0.26 |
|  | Other | 0.33 | 0.25 | 0.22 | 0.21 | -0.04 | 0.02 | | 0.48 | 0.28 |
| Tumor location | Right colon set to 0 as baseline hazard | | | | | | | | | |
|  | Left colon | 0.05 | 0.00 | -0.02 | 0.03 | -0.04 | -0.01 | | 0.00 | -0.03 |
|  | Rectum | 0.21 | 0.18 | 0.05 | 0.11 | 0.02 | -0.05 | | 0.08 | 0.02 |
| T stage | T1 set to 0 as baseline hazard | | | | | | | | | |
|  | T2 | 0.04 | 0.21 | 0.21 | 0.15 | 0.30 | 0.12 | | 0.23 | 0.05 |
|  | T3 | 0.47 | 0.46 | 0.52 | 0.48 | 0.46 | 0.37 | | 0.46 | 0.26 |
|  | T4a | 1.15 | 0.86 | 0.87 | 0.85 | 0.78 | 0.70 | | 0.93 | 0.63 |
|  | T4b | 1.11 | 1.38 | 1.24 | 1.18 | 1.35 | 1.17 | | 1.08 | 1.18 |
| N stage | N0 set to 0 as baseline hazard | | | | | | | | | |
|  | N1a | 0.23 | 0.36 | 0.24 | 0.30 | 0.33 | 0.12 | | 0.29 | 0.32 |
|  | N1b | 0.25 | 0.47 | 0.34 | 0.43 | 0.35 | 0.29 | | 0.41 | 0.44 |
|  | N1c | -0.10 | -13.98 | 0.59 | 0.22 | -0.03 | 0.62 | | 0.45 | 0.79 |
|  | N1nos | 0.60 | -0.15 | 0.27 | 0.26 | 0.48 | 0.34 | | 0.06 | 0.24 |
|  | N2a | 0.43 | 0.63 | 0.47 | 0.56 | 0.54 | 0.38 | | 0.41 | 0.45 |
|  | N2b | 0.67 | 0.41 | 0.46 | 0.75 | 0.73 | 0.33 | | 0.51 | 0.71 |
|  | N2nos | NA | NA | 2.58 | -9.70 | -10.90 | 2.32 | | NA | NA |
| Tumor size | Mean value as baseline hazard | 0.00 | 0.00 | 0.00 | 0.00 | 0.00 | 0.00 | | 0.00 | 0.00 |
| EOD10_PN | Mean value as baseline hazard | 0.05 | 0.06 | 0.05 | 0.03 | 0.04 | 0.06 | | 0.05 | 0.05 |

**Table S4. Regression coefficients of covariates of the statistical model based on a Cox model with mixed effects.**

| Predictor | Levels | Coef | se(coef) | Chisq | p |
| --- | --- | --- | --- | --- | --- |
| Age at diagnosis | Continuous variable | 0.05 | 0.00 | 9856.07 | 0.00 |
| Gender | Male set to 0 as baseline hazard | |  |  |  |
|  | Female | -0.19 | 0.01 | 243.88 | 0.00 |
| Grade | Well-differentiated group set to 0 as baseline hazard | | | |  |
|  | Moderately differentiated | 0.06 | 0.02 | 7.04 | 0.01 |
|  | Poorly differentiated | 0.20 | 0.03 | 55.43 | 0.00 |
|  | Undifferentiated | 0.30 | 0.05 | 43.88 | 0.00 |
| Histology | Adenocarcinoma group set to 0 as baseline hazard | | | |  |
|  | Mucinous adenocarcinoma | 0.06 | 0.02 | 10.55 | 0.00 |
|  | Papillary adenocarcinoma | 0.02 | 0.02 | 0.98 | 0.32 |
|  | Adenoma. In Adenoma. Polyp | 0.01 | 0.04 | 0.09 | 0.77 |
|  | Signet ring cell carcinoma | 0.36 | 0.05 | 49.79 | 0.00 |
|  | Other | 0.20 | 0.04 | 22.05 | 0.00 |
| Tumor location | Right colon set to 0 as baseline hazard | | |  |  |
|  | Left colon | 0.01 | 0.01 | 0.57 | 0.45 |
|  | Rectum | 0.13 | 0.02 | 64.98 | 0.00 |
| T stage | T1 set to 0 as baseline hazard | |  |  |  |
|  | T2 | 0.17 | 0.03 | 30.60 | 0.00 |
|  | T3 | 0.44 | 0.03 | 229.68 | 0.00 |
|  | T4a | 0.85 | 0.03 | 590.81 | 0.00 |
|  | T4b | 1.18 | 0.04 | 1076.67 | 0.00 |
| N stage | N0 set to 0 as baseline hazard | |  |  |  |
|  | N1a | 0.26 | 0.02 | 177.32 | 0.00 |
|  | N1b | 0.37 | 0.02 | 355.84 | 0.00 |
|  | N1c | 0.30 | 0.10 | 8.20 | 0.00 |
|  | N1nos | 0.25 | 0.04 | 35.05 | 0.00 |
|  | N2a | 0.49 | 0.02 | 418.23 | 0.00 |
|  | N2b | 0.61 | 0.04 | 270.73 | 0.00 |
|  | N2nos | -0.26 | 0.58 | 0.20 | 0.66 |
| Tumor size | Size of tumor | 0.00 | 0.00 | 48.95 | 0.00 |
| EOD10_PN | Number of positive regional lymph nodes | 0.05 | 0.00 | 339.01 | 0.00 |
| Spatial cluster | Frailty term | NA | NA | 248.85 | 0.00 |

**Table S5. Test result of proportional hazards assumption for the Cox model with mixed effects.**

| Predictor | Levels | Rho | Chisq | p |
| --- | --- | --- | --- | --- |
| Age at diagnosis | Continuous variable | -0.02 | 20.14 | 0.00 |
| Gender | Male set to 0 as baseline hazard |  |  |  |
|  | Female | 0.00 | 0.43 | 0.51 |
| Grade | Well-differentiated group set to 0 as baseline hazard |  |  |  |
|  | Moderately differentiated | -0.01 | 3.12 | 0.08 |
|  | Poorly differentiated | -0.04 | 53.84 | 0.00 |
|  | Undifferentiated | -0.03 | 19.94 | 0.00 |
| Histology | Adenocarcinoma group set to 0 as baseline hazard |  |  |  |
|  | Mucinous adenocarcinoma | 0.01 | 0.73 | 0.39 |
|  | Papillary adenocarcinoma | 0.01 | 1.84 | 0.18 |
|  | Adenoma. In Adenoma. Polyp | 0.01 | 1.62 | 0.20 |
|  | Signet ring cell carcinoma | 0.01 | 1.06 | 0.30 |
|  | Other | -0.01 | 1.46 | 0.23 |
| Tumor location | Right colon set to 0 as baseline hazard |  |  |  |
|  | Left colon | 0.01 | 4.74 | 0.03 |
|  | Rectum | 0.03 | 30.50 | 0.00 |
| T stage | T1 set to 0 as baseline hazard |  |  |  |
|  | T2 | 0.01 | 2.08 | 0.15 |
|  | T3 | 0.00 | 0.65 | 0.42 |
|  | T4a | -0.01 | 2.45 | 0.12 |
|  | T4b | -0.01 | 5.41 | 0.02 |
| N stage | N0 set to 0 as baseline hazard |  |  |  |
|  | N1a | -0.02 | 6.83 | 0.01 |
|  | N1b | -0.02 | 7.05 | 0.01 |
|  | N1c | 0.00 | 0.46 | 0.50 |
|  | N1nos | -0.01 | 4.52 | 0.03 |
|  | N2a | -0.02 | 11.53 | 0.00 |
|  | N2b | -0.03 | 16.45 | 0.00 |
|  | N2nos | 0.00 | 0.21 | 0.65 |
| Tumor size | Size of tumor | -0.04 | 33.10 | 0.00 |
| EOD10_PN | Number of positive regional lymph nodes | 0.02 | 5.36 | 0.02 |
| GLOBAL |  | NA | 421.72 | 0.00 |

Table S6. The AIC for different distributions of the AFT model.

| Distribution |  | AIC | Log-likelihood |
| --- | --- | --- | --- |
| log-logistic |  | 319672.6 | -159809.3 |
| weibull |  | 319707.3 | -159823.7 |
| exponential |  | 319721.2 | -159833.6 |
| log-normal |  | 320882.2 | -160414.1 |
| gaussian |  | 345036.6 | -172491.3 |
| logistic |  | 347314.9 | -173630.5 |


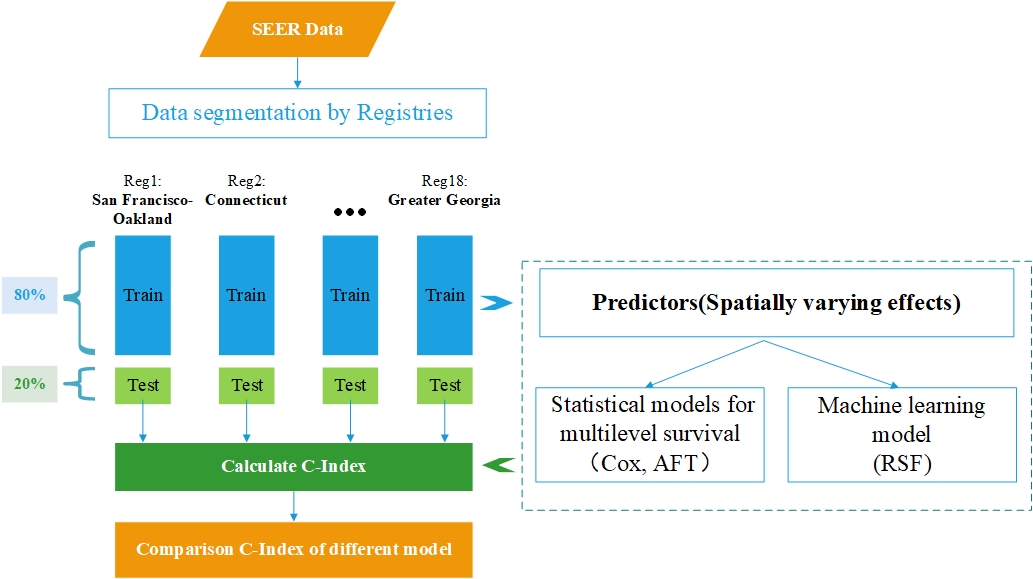


Fig. S1. The specific model process used for the machine learning model and the statistical model. First, we divided the SEER research data into groups according to geographical registry. Second, we subdivided the datasets of each geographical registry into training sets (80%) and test sets (20%). Third, we aggregated the training datasets for all registries and fitted the data to both models considering the following predictors: age at diagnosis, gender, grade, histology, tumor location, T stage, N stage, tumor size and number of positive regional lymph nodes. For the statistical model, except for tumor size and the number of positive regional lymph nodes, all the predictors were categorized based on Table 1, and random effects on the different geographical registries were considered. For the machine learning model, age at diagnosis, tumor size and the number of positive regional lymph nodes were considered continuous predictors, and the geographical registry was considered an individual predictor. The accelerated failure time (AFT) approach is an alternative strategy to analyze time-to-event data and can be suitable even when hazards are not proportional.
